# Efficacy and Tolerance of Single-dose Azithromycin for Treatment of Chlamydial Cervicitis During Pregnancy

**DOI:** 10.1155/S1064744995000597

**Published:** 1995

**Authors:** Joseph M. Miller

**Affiliations:** Department of Obstetrics and Gynecology Louisiana State University Medical Center 1542 Tulane Avenue New Orleans LA 70112-2822 USA

## Abstract

*Objective:* The intent of this study was to determine the efficacy and tolerance of single-dose oral azithromycin in the treatment of pregnant women with endocervical chlamydial carriage.

*Methods:* A retrospective review of clinic records over a two-year period identified pregnant patients treated with a single 1-g dose of azithromycin for chlamydial carriage. The side effects and subsequent chlamydial carriage (test of cure) were noted.

*Results:* A total of 146 pregnant women treated with azithromycin was reviewed. A cure rate of 96% was found. Side effects were reported in 5%.

*Conclusions:* A single 1-g oral dose of azithromycin is effective for the treatment of chlamydia and is well tolerated in pregnant women.


Infectious Diseases in Obstetrics and Gynecology 3:189–192 (1995)
© 1996 Wiley-Liss, Inc.

### Efficacy and Tolerance of Single-Dose Azithromycin for Treatment of Chlamydial Cervicitis During Pregnancy

**Joseph M. Miller, Jr.**

*Department of Obstetrics and Gynecology, Louisiana State University Medical Center, New Orleans, LA*

ABSTRACT

***Objective:*** **The intent of this study was to determine the efficacy and tolerance of single-dose oral azithromycin in the treatment of pregnant women with endocervical chlamydial carriage.**

***Methods:*** **A retrospective review of clinic records over a two-year period identified pregnant patients treated with a single 1-g dose of azithromycin for chlamydial carriage. The side effects and subsequent chlamydial carriage (test of cure) were noted.**

***Results:*** **A total of 146 pregnant women treated with azithromycin was reviewed. A cure rate of 96% was found. Side effects were reported in 5%.**

***Conclusions:*** **A single 1-g oral dose of azithromycin is effective for the treatment of chlamydia and is well tolerated in pregnant women.** © 1996 Wiley-Liss, Inc.

KEY WORDS
Chlamydia, prenatal care, STD screening

*Chlamydia trachomatis* carriage in pregnancy poses concerns for the patient, her unborn child, and her sexual partner. Erythromycin is now recommended,[1] but it is associated with significant gastrointestinal side effects.[2,3] Noncompliance is common, occurring in nearly 40% of patients.[4] A new drug, azithromycin, in a single 1-g dose, has been approved for the treatment of chlamydial cervicitis in nonpregnant individuals. A small prospective study in pregnant women showed that azithromycin was associated with cure rates similar to those of erythromycin but was significantly better tolerated.[5] We elected to use azithromycin preferentially because of potential enhanced compliance, reduced side effects, and excellent cure rate. The only alternate drug freely available was erythromycin. Presented is our experience with azithromycin's efficacy and tolerance in over 100 pregnant patients followed in our practice.

### SUBJECTS AND METHODS

The patient population attended a community-based prenatal and family planning clinic adjacent to 2 low-income housing projects between January 1, 1993, and December 31, 1994. Surveillance data previously identified chlamydial cervicitis as being present in greater that 20% of the patients attending this clinic.[6] The patients included in this report were identified by an audit of all clinic records. Only the patients with follow-up information about side effects were included. Five patients lacking such information were excluded. Patient characteristics are given in Table 1.

Cervical swabs were analyzed by direct DNA assay (Gen-Probe, San Diego, CA), routinely performed on all obstetric patients at their initial visits and again at 34 weeks of gestation. This test has a sensitivity of 76–96% and a specificity of 98%.[7,8] The pregnant women with positive chlamydial

**Address correspondence/reprint requests to Dr Joseph M. Miller, Jr., Department of Obstetrics and Gynecology, Louisiana State University Medical Center, 1542 Tulane Avenue, New Orleans, LA 70112-2822.**

Received June 27, 1995
Accepted October 19, 1995
Clinical Study

TABLE 1. Population characteristics

| Characteristic | No. of patients |
|---|---|
| Ethnic background | |
| Afro-American | 142 |
| Caucasian | 2 |
| Hispanic | 2 |
| Parity | |
| 0 | 101 |
| 1 | 28 |
| 2 | 9 |
| ≥3 | 8 |
| Maternal age in years | |
| ≤15 | 10 |
| 16–17 | 37 |
| 18–19 | 49 |
| 20–24 | 34 |
| 25–30 | 12 |
| >30 | 4 |

screens were offered treatment with 1 g of azithromycin on site and were observed for 30 min after the oral medication was administered. The patients were questioned later as to the occurrence of side effects, including nausea, vomiting, diarrhea, and abdominal pain. This information was obtained by patient recall at a subsequent clinic visit; diaries were not kept. The patients were instructed to abstain from intercourse until their sexual partners had completed treatment. Their partners were referred to a sexually transmitted disease (STD) clinic available to all patients. The occurrence of chlamydia as an STD was reported to the State Health Department. A test of cure was generally obtained 10–14 days after treatment. The patient's age, race, parity, and pregnancy outcome (gestational age and birth weight) were obtained from a chart review. The presence of a positive test for other STDs (Venereal Disease Research Laboratory reaction [VDRL], human immune deficiency virus antibody [HIV] confirmed by western blot, gonorrhea by Gen-probe, hepatitis B surface antigen, and human papillomavirus by pap smear) at any time during pregnancy was also recorded. These tests were obtained at the initial visit. The VDRL and both chlamydia and gonorrhea assays by Gen-probe were repeated at 34 weeks of gestation. Patients with signs or symptoms of genital herpes were cultured; otherwise, cultures were not routinely obtained.

### RESULTS

The treated patients were primarily young. Ten patients were ≤15 years, 37 were 16–17 years, 49 were 18–19, 34 were 20–24, 12 were 28–30, and 4 were >30 years. There were 101 nulliparas; 28 had previously delivered 1 child, 9 had delivered 2 children, and 8 had delivered 3 or more children. One hundred forty-two patients were Afro-American, 2 were Hispanic, and 2 were Caucasian. Two patients had twins. The initial positive screens for chlamydia were reported and the patients were treated: 35/146 in the first trimester, 71 in the second trimester, and 40 in the third trimester.

Other STDs were identified. A carriage of *Neisseria gonorrhoeae* was found in 33 patients. Four had positive herpes cultures during pregnancy. Two patients had positive VDRLs. One patient was positive for hepatitis B surface antigen. Another patient was HIV positive. Four patients had evidence of human papillomavirus on their pap smears. Twenty-two of the treated patients were chlamydia negative at the time of their initial obstetric evaluations, but subsequently were positive.

Azithromycin was generally well tolerated, with only 8/146 patients experiencing side effects. Seven patients experienced nausea. Six of them vomited within 2 h of treatment; the seventh complained of abdominal pain as well. One patient developed diarrhea. Cefixime, 400 mg, was also administered subsequent to azithromycin to 22 patients with positive tests for gonorrhea 30–60 min after the initial treatment for chlamydia. Other antibiotics, were initiated several hours later, including metronidazole in 9 patients (4 for bacterial vaginosis and 5 for trichomonas); clindamycin vaginal cream for 2 patients with bacterial vaginosis; and, for urinary-tract infections, ampicillin for 3 patients and nitrofurodantoin for 1 patient. Of the 6 patients who vomited, 1 received metronidazole and another cefixime within 2 h, but before vomiting. Two of the 6 patients who vomited were empirically retreated with azithromycin; all 6 had negative tests of cure.

The treatment was successful in 132/138 patients who had tests of cure. Eight patients either delivered (n = 1) or were lost to follow-up (n = 7). Reinfection, defined by a subsequent positive test after the test of cure was negative, was found in 15/67 patients who were retested during the same pregnancy after the negative test of cure. Retreatment with azithromycin was routinely done for reinfection and was effective in all 10 for whom tests of cure were subsequently obtained. The retreatment was not included in the treatment efficacy or side effect data.

The 6 patients who failed therapy had tests of cure obtained 19, 35, 50, 51, 61, and 67 days after treatment. Reinfection rather than treatment failure may well have been present.

Late miscarriage (at 15, 16, and 18 weeks of gestation) occurred in 3 patients. A review of the records indicated that 2 delivered 4 days after treatment, 1 for an incompetent cervix. A third patient delivered 15 days after treatment. A fourth patient with spotting prior to treatment aborted 4 days later while in the first trimester. One patient underwent a pregnancy termination. Delivery before 37 weeks of gestation occurred in 19/125 patients. The delivery information was unavailable in 16. The birth weight was 1,500–2,499 g for 15 infants and <1,500 g in 1. The pregnancy outcomes were reflective of the patient population served.[6]

### DISCUSSION

A single 1-g dose of azithromycin was found to be an effective treatment of *C. trachomatis* endocervical carriage in pregnant women, supporting a smaller study in pregnant women[5] and a larger one in nonpregnant women.[9] The side effects, which did not vary substantially from those previously reported, were less common than those reported with erythromycin use in pregnancy.[2] Single-dose treatment, which is both effective and well tolerated, holds great promise for patients who may already be experiencing nausea while pregnant or for patients who maybe noncompliant with antibiotics. Noncompliance occurs more often among younger patients and those experiencing side effects.[4] Currently, amoxicillin is held to be a reasonable alternative treatment for chlamydial endocervical carriage, with a cure rate of 85–98%.[10,11] We believe the cure rate with azithromycin of 96%, together with the low rate of side effects, ease of administration, and absence of discontinuance by the patient, is sufficient to select it over the alternate drugs of amoxicillin and erythromycin for the population served by our program. There is little risk to the fetus from treatment with this class B drug (Pfizer Labs prescribing information, February, 1992). Class B drugs have no evidence of risk in humans: either animal findings show risk but human findings do not or, in the absence of adequate human studies, animal findings are negative.[12] However, sufficient follow-up with azithromycin, which is yet to be published, would provide a high level of reassurance.

Reinfection was a frequent occurrence in our study. Appropriate follow-up studies for chlamydia appear warranted in our population. Twenty-two percent of the treated and "cured" patients who were retested demonstrated chlamydia carriage later in the same pregnancy. The population characteristics of our study patients, being primarily young Afro-Americans residing in an urban setting, are recognized risk factors for recurrent infections in women.[13] Instructions to the patient may need to reemphasize the importance of condoms in the prevention of STDs[1] as well as appropriate evaluation, treatment, and follow-up of sexual partners.

### REFERENCES

1. Sexually transmitted disease treatment guidelines. MMWR 42:52, 1993.
2. Magat AH, Alger LS, Nagey DA, Hatch V, Lovchik JC. Double-blind randomized study comparing amoxicillin and erythromycin for the treatment of *Chlamydia trachomatis* in pregnancy. Obstet Gynecol 81:745–749, 1993.
3. Linnemann CC Jr, Heaton CL, Ritchey M. Treatment of *Chlamydia trachomatis* infections: Comparison of 1- and 2-g doses of erythromycin daily for seven days. Sex Transm Dis 14:102–106, 1987.
4. Katz BP, Zwichal BW, Caine MA, Jones RB. Compliance with antibiotic therapy for *Chlamydia trachomatis* and *Neisseria gonorrhoeae*. Sex Transm Dis 19;351–354, 1992.
5. Bush MR, Rosa C. Azithromycin and erythromycin in the treatment of cervical chlamydial infection during pregnancy. Obstet Gynecol 84:61–63, 1994.
6. Boudreaux M, Miller JM Jr, Wightkin J, Martin S, Mather F. Can nurse managed high risk pregnancy care work? (in press).
7. Blanding J, Hirsch L, Stranton N, et al. Comparison of the Clearview Chlamydia, the PACE 2 assay, and culture for the detection of *Chlamydia trachomatis* from cervical specimens in a low-prevalence population. J Clin Microbiol 31:1622–1625, 1993.
8. Warren R, Dwyer B, Plackett M, Pettit K, Rivi N, Baker A. Compositive evaluation of detection assays for *Chlamydia trachomatis*. J Clin Microbiol 31:1663–1666, 1993.
9. Martin DH, Mroczkowski TF, Dalu ZA, et al. A controlled trial of a single dose of azithromycin for the treatment of chlamydial urethritis and cervicitis. N Engl J Med 327:921–925, 1992.
10. Silverman NS, Sullivan M, Hochman M, Womack M,

Jungkind DL. A randomized, prospective trial comparing amoxicillin and erythromycin for the treatment of *Chlamydia trachomatis* in pregnancy. Am J Obstet Gynecol 170:829–832, 1994.
11. Cromblehome WR, Schechter J, Grossman M, Landers DV, Sweet RL. Amoxicillin therapy for *Chlamydia trachomatis* in pregnancy. Obstet Gynecol 75:752–756, 1990.
12. FDA Drug Bull 12:24–25, 1982.
13. Hillis SD, Nakashima A, Marchbanks PA, Addiss DG, Davis JP. Risk factors for recurrent *Chlamydia trachomatis* infections in women. Am J Obstet Gynecol 170:801–806, 1994.
